# Oral Cancer Stem Cells: Therapeutic Implications and Challenges

**DOI:** 10.3389/froh.2021.685236

**Published:** 2021-07-21

**Authors:** Linah A. Shahoumi

**Affiliations:** Department of Oral Biology and Diagnostic Sciences, Dental College of Georgia, Augusta University, Augusta, GA, United States

**Keywords:** cancer stem cells, head and neck, squamous cell carcinoma, self-renewal, tumor relapse, targeted therapy

## Abstract

Head and neck squamous cell carcinoma (HNSCC) is currently one of the 10 most common malignancies worldwide, characterized by a biologically highly diverse group of tumors with non-specific biomarkers and poor prognosis. The incidence rate of HNSCC varies widely throughout the world, with an evident prevalence in developing countries such as those in Southeast Asia and Southern Africa. Tumor relapse and metastasis following traditional treatment remain major clinical problems in oral cancer management. Current evidence suggests that therapeutic resistance and metastasis of cancer are mainly driven by a unique subpopulation of tumor cells, termed cancer stem cells (CSCs), or cancer-initiating cells (CICs), which are characterized by their capacity for self-renewal, maintenance of stemness and increased tumorigenicity. Thus, more understanding of the molecular mechanisms of CSCs and their behavior may help in developing effective therapeutic interventions that inhibit tumor growth and progression. This review provides an overview of the main signaling cascades in CSCs that drive tumor repropagation and metastasis in oral cancer, with a focus on squamous cell carcinoma. Other oral non-SCC tumors, including melanoma and malignant salivary gland tumors, will also be considered. In addition, this review discusses some of the CSC-targeted therapeutic strategies that have been employed to combat disease progression, and the challenges of targeting CSCs, with the aim of improving the clinical outcomes for patients with oral malignancies. Targeting of CSCs in head and neck cancer (HNC) represents a promising approach to improve disease outcome. Some CSC-targeted therapies have already been proven to be successful in pre-clinical studies and they are now being tested in clinical trials, mainly in combination with conventional treatment regimens. However, some studies revealed that CSCs may not be the only players that control disease relapse and progression of HNC. Further, clinical research studying a combination of therapies targeted against head and neck CSCs may provide significant advances.

## Introduction

Head and neck cancer is a heterogenous group of tumors which mainly arise in the oral cavity, oropharynx, hypopharynx, salivary glands, paranasal sinuses and larynx [1]. HNC is among the most common cancers worldwide, with a high prevalence in Southeast Asia, Brazil, and Southern Africa [2]. Squamous cell carcinomas make up the majority of HNC which have an incidence of around 630,000 new cases per year worldwide, with almost 10,000 deaths in the United States annually [3, 4]. Lifestyle behaviors such as drinking alcohol, use of tobacco, and chewing betel quid are the most common risk factors associated with HNSCC [5]. Despite recent considerable advances in the therapeutic repertoire for oral cancer, the median overall survival for patients with metastatic or recurrent HNC remains <1 year [6]. Resistance to chemotherapeutic and biologic agents is responsible for the failure of many current therapeutic approaches. Accumulating evidence suggests that the molecular complexity, intratumoral heterogeneity, and presence of CSCs are responsible for local recurrence, metastatic spread, and treatment resistance in various types of cancer [7]. Intratumoral heterogeneity may be the consequence of genetic alterations, epigenetic modification, and changes in cell properties and behavior [8]. CSCs are a pluripotent subpopulation of cells in the tumor that have attributes of self-renewal, tumor initiation, differentiation, migration, and metastasis [9–11]. This subpopulation has been identified in several solid tumors, including HNC, and it shows certain characteristics that give it the ability to re-create entire heterogeneous populations of a tumor posttreatment, causing tumor recurrence and metastasis [12]. This review will discuss the characterization and molecular regulation of CSCs that drive tumor progression and metastasis in oral cancer, with a focus on HNSCC, highlighting the role of CSCs in treatment failure. Furthermore, this review will outline CSC-targeted strategies, as well as challenges of targeting CSCs, with the aim of making a potential tangible difference in clinical outcome for HNC patients.

## Characterization of HNSCC Cancer Stem Cells

**Figure 1:**
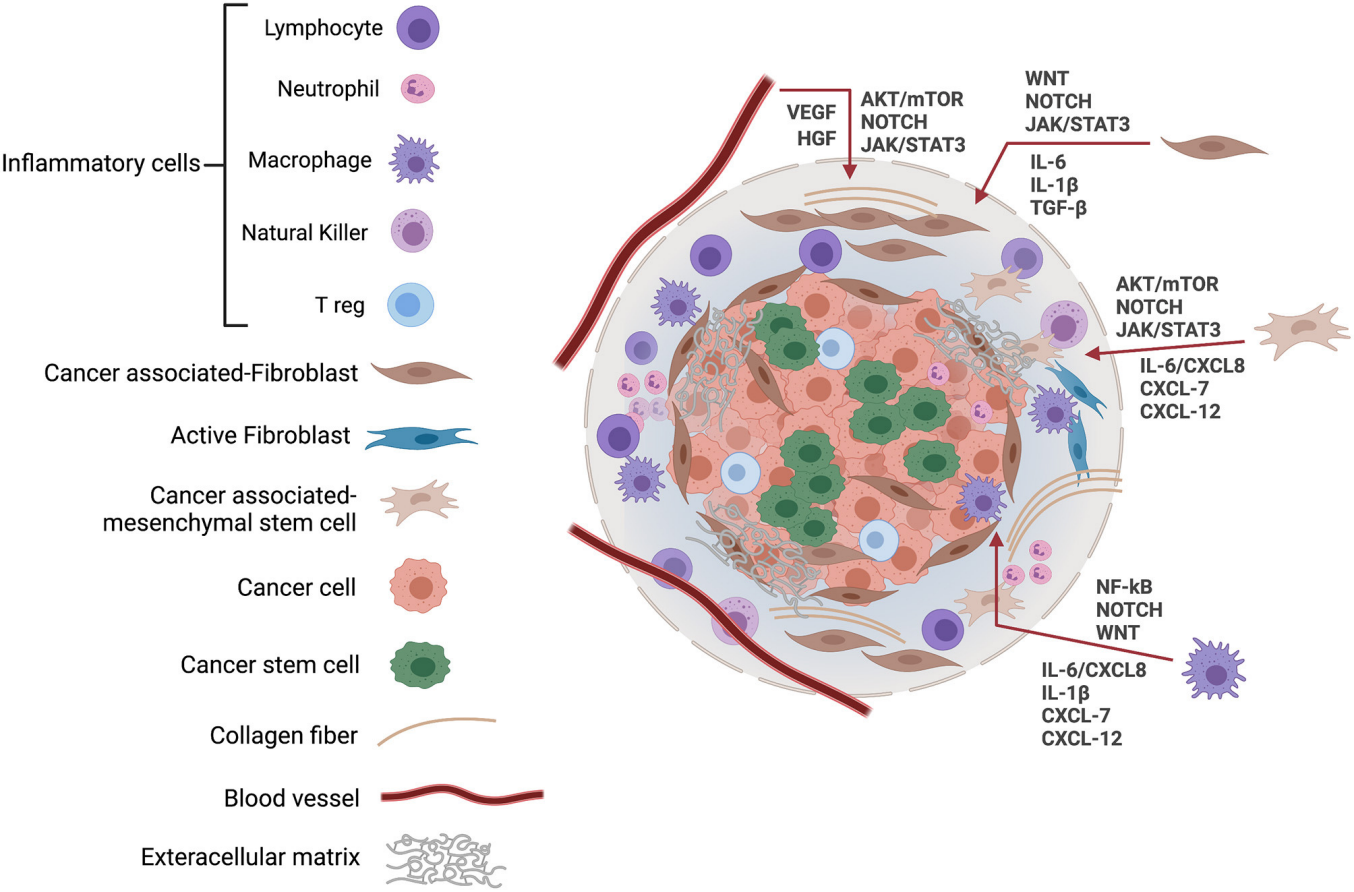
The cancer stem cell microenvironment. The niche or tumor microenvironment is essential for maintenance of stemness, and this also applies to CSCs where cell–cell interactions within the niche are required to support the role of CSCs in cancer initiation and progression. The CSC microenvironment also contributes to CSCs' resistance to drugs and other cancer therapies, thereby, promoting cancer recurrence. The tumor tissue microenvironment is composed of a variety of cells, including tumor cells, cancer stem cells, inflammatory cells, and cancer-associated fibroblasts, along with blood vessels and extracellular matrix. In response to hypoxic stress and matrix, CSCs induce growth factors and cytokines including IL-6, CXCL8, and VEGF to regulate their growth *via* EGFR, NOTCH, WNT, and other signaling cascades. JAK, Janus kinases; STAT, signal transducer, and activator of transcription; VEGF, vascular endothelial growth factor; HGF, hepatocyte growth factor; mTOR, mammalian target of rapamycin; IL, Interleukin; CXCL, CXC-motif chemokine ligand.

CSCs interact with transformed cells and other stromal cells within the tumor microenvironment through adhesion molecules and paracrine factors. These microenvironmental interactions promote the differentiation of CSCs, enhance angiogenesis, recruit immune and stromal cells, and promote tumor invasion and metastasis [13] (Figure 1). CSCs were first identified in leukemia [14], and subsequently in various types of cancer, including tumors in brain [15], lung [16], colon [17], breast [18], and HNSCC [19]. CSCs are believed to have multiple unique features including the potential for differentiation and self-renewal that make them crucial for tumorigenesis [20], and potentially offer a novel area of study for developing more effective treatments for HNSCC. It was previously reported that CSCs in HNSCC play a vital role in initiation, invasion, and progression as well as resistance to chemo/radiotherapy, and they are responsible for recurrence and metastasis [21]. The literature reveals that several different markers have been used to identify CSCs in HNSCC. These markers are not only employed for selective CSC isolation but are also involved in regulating multiple biological functions of CSCs, including cell proliferation, invasion, self-renewal and survival, effectively promoting tumor progression, and recurrence.

### CSC Metabolic and Cell-Surface Markers in HNSCC

CSCs in HNSCC were initially identified and isolated by their high levels of expression of the hyaluronan receptor CD44 [19, 22]. CD44 is a type I transmembrane glycoprotein involved in intercellular interactions, cell migration, adhesion, and angiogenesis [23–25]. The extracellular domain of CD44 can interact with hyaluronic acid and other ligands, including cytokines, and matrix metalloproteinases [26, 27]. CD44 plays a role in cell proliferation, survival and tumorigenesis through activation of multiple tyrosine kinase-induced pathways including epidermal growth factor receptor (EGFR), Src/focal adhesion kinase (FAK), and hepatocyte growth factor receptor (MET) [28–30]. Importantly, clinical reports indicated that CD44 expression is associated with local recurrence, regional lymph node metastasis, perineural invasion, and poorer survival rate in oral squamous cell carcinoma (OSCC) [31–34], indicating its vital role in tumor recurrence and metastasis of HNSCC. In addition, Biddle et al. [35] examined the therapeutic resistance of heterogeneous CSC subpopulations that express high levels of CD44 and found that these subpopulations exhibit high plasticity and increased therapeutic resistance. CD10, a type II integral membrane protein also known as neutral endopeptidase 24.11, is another potential marker for HNSCC stem cells. Previous studies reported a role for CD10 in the differentiation and growth of neoplastic cells and that its expression was associated with tumor size, histological grade of malignancy, local recurrence, and therapeutic resistance in HNSCC [36, 37]. Moreover, HNSCC patients with high CD10 expression had significantly poorer overall survival (OS) [38]. Another well-known cell surface marker for isolation of human malignant tissue stem cells is CD133. CD133 is a transmembrane glycoprotein which has been used as a marker to identify CSCs derived from primary solid tumors [16, 39, 40]. Previously, CD133^+^ cell populations were found to have higher cell viability, migratory and invasive capability, colony forming ability as well as drug resistance compared with CD133^−^ populations [41–43]. Moreover, CD133 expression has been used as a prognostic marker of survival in HNSCC and is negatively correlated with clinical outcome in these patients [44]. However, other studies reported no CD133 expression in freshly prepared HNSCC patient samples and no correlation was detected between CD133 expression and differentiation of carcinoma cells, or prognosis [45–47]. Therefore, the value of CD133 as a marker for HNSCC CSCs remains unclear and may need to be re-evaluated.

HNSCC CSCs also have elevated activity of aldehyde dehydrogenase (ALDH), an intracellular enzyme that metabolizes reactive aldehydes producedfrom alcohol and chemotherapeutic compounds into non-reactive acids [48]. Mutations and altered expression of various ALDH genes are implicated in multiple cancers including HNSCC [49–51]. ALDH1 was found to colocalize with other CSC-related markers, including Snail and MMP-9, and induce epithelial to mesenchymal transition (EMT)-related genes [49, 52]. ALDH1^+^ cells from HNSCC patients display a more tumorigenic phenotype, self-renewal and stemness properties, and also resistance to radiotherapy and chemotherapy [49]. Furthermore, High levels of ALDH1 in patient samples have been correlated with poor prognosis in HNSCC [53, 54]. In contrast, another clinical report investigated CSC populations in 74 locally advanced HNSCCs from an equal number of patients, treated with accelerated platinum-based radiotherapy, and revealed that high expression of ALDH1 and lack of CD44 expression in CSCs was associated with better radiotherapy outcome and favorable prognosis [55]. These contradictory results suggested that, in some tumors, terminally differentiated cancer cells may retain expression of stem cell markers. Although, tremendous efforts have been made to understand CSC-associated molecules and to find an optimal marker for CSCs in HNSCC, challenges still remain because of the heterogeneity of CSCs and the absence of one marker does not necessary mean that cells do not possess characteristics of stemness.

### Transcriptional Factors of HNSCC CSCs

HNSCC CSCs express same proteins involved in the core network that regulates embryonic stem cells (ESCs). In particular, Octamer-binding transcription factor 4 (OCT4; POU5F1), Sex determining region Y-box 2 (SOX2) and Nanog Homeobox (NANOG) are highly enriched in both CSCs and ESCs [56, 57]. These transcription factors are considered master transcriptional regulators that orchestrate stemness properties including self-renewal, angiogenesis, migration, and resistance to apoptosis [58, 59]. OCT4 has three domains, including the POU domain (DNA binding domain) which is essential in early embryogenesis and for maintenance of pluripotent cells during embryonic development [60]. OCT4 has been reported to be overexpressed in various malignancies, including HNSCC, lung, breast, liver, and ovarian cancer [58, 61–64]. It has been suggested that OCT4 plays a crucial role in regulating EMT by increasing the expression of the mesenchymal marker N-cadherin and the transcription factor SNAI2 (Slug) to promote tumor progression and metastasis [10]. Moreover, OCT4 overexpression was observed in tumor cells metastatic to lymph nodes and also in recurrent tumors from oral squamous cell carcinoma (OSCC) patients, indicating that OCT4 may be a potential marker of recurrence and metastasis in HNSCC [10]. Furthermore, accumulating evidence indicates that OCT4 is correlated with poor survival and it has been suggested as an independent prognostic marker of HNSCC progression [58, 65]. SOX2 is a high-mobility group (HMG) domain-containing transcription factor pivotal for pluripotent cell development [66]. Indeed, SOX2 is known to complex with OCT4 which binds to important regulatory elements of POU5F1, the gene encoding OCT4, to maintain the expression of essential transcription factors in ESCs through autoregulatory and multicomponent loop network motifs [67]. In addition, SOX2 is involved in many processes important in oncogenesis including cell proliferation, stemness, migration, invasion, tumorigenesis, and chemoresistance [68]. A genomic copy number gain at the SOX2 locus results in an increase in SOX2 transcriptional activity, and this is reported to be critical for HNSCC initiation and progression [69]. Moreover, SOX2 collaborates with other oncoproteins such as signal transducer and activator of transcription 3 (STAT3) to initiate and induce SCC [70]. Overexpression of SOX2 promotes cell proliferation *via* cyclin B1 upregulation and enhances stem cell properties [71]. Importantly, SOX2 expression levels correlate positively with radio-chemoresistance and poor prognosis in HNSCC patients [68, 71]. On the other hand, it has been suggested that both OCT4 and SOX2 expression are associated with early tumor stage and better disease-free survival, raising the possibility that both SOX2 and OCT4 might not be the best targets to eradicate tumor relapse and progression [57]. Of interest, the reduction of SOX2 and OCT4 expression in advanced-stage HNSCC, as reported in a study by Fu et al. [57], suggests that overexpression of SOX2 seen in early lesions might decrease gradually during tumor progression. However, whether the molecular mechanisms regulating SOX2 and OCT4 are associated with a favorable prognosis in HNSCC is still unknown and was not investigated in this study. A key downstream target of OCT4 and SOX2 is NANOG, which is a variant homeobox transcription factor widely recognized as a marker for stemness [72, 73]. Functionally, NANOG is involved in blocking differentiation to maintain ESC pluripotency and has been shown to be overexpressed in various cancers including HNSCC [58, 74]. In HNSCC, NANOG can promote cell proliferation, invasion, and colony formation of the CSC population *via* its phosphorylation at T200 and T280 by protein kinase C epsilon (PKCε) [75]. Recently, evidence has been provided to suggest that high expression of NANOG promotes EMT, the acquisition of CSC properties, and enhances radiotherapy resistance in HNSCC [76]. Moreover, elevated NANOG expression was reported as a prognostic biomarker for OS in HNSCC and shown to correlate with poor differentiation and chemoresistance [77]. These findings indicate that NANOG may be a novel target for elimination of CSCs in HNSCC. B lymphoma Mo-MLV insertion region 1 homolog (BMI1) is a core component of the polycomb repressive complex 1 (PRC1) that mediates gene silencing *via* monoubiquitination of histone H2A. BMI1 is strongly linked to self-renewal and has been implicated in maintaining the stem cell pool of several tissues [78]. The N- terminal ring-finger domain of BMI1 is required for the activation of telomerase reverse transcriptase (TERT) transcription and immortalization of epithelial cells [79]. BMI1 is among the most studied CSC markers in HNSCC [80]. High expression of BMI1 in cancer was related to EMT and involved in the invasion and progression of tongue squamous cell carcinoma [81]. Several studies have reported that BMI1 is abnormally expressed in HNSCC and correlated with advanced tumor stages, drug resistance, and poor prognosis [19, 34, 82]. However, other studies could not predict survival from BMI1 expression [83, 84]. The reason of these contradictory results could be the limited scope to consider heterogeneity in CSCs as different types or states of CSCs promoting disease progression and others slowing it down.

### Signaling Pathways Utilized by CSCs in HNSCC

Multiple pathways are involved in the regulation of normal stem cell differentiation and self-renewal. The NOTCH and Sonic hedgehog (SHH) pathways have been implicated in the regulation of differentiation and patterning of numerous organ systems [85]. SHH, a secreted glycoprotein, activates signaling in target cells by binding to the transmembrane receptor Patched 1 (PTCH1) which unleashes Smoothened (SMO) to trigger a series of intracellular pathways that induce the translocation of the transcription factor glioma-associated oncogene homolog 1 (GLI1) into the nucleus [86] where it induces the transcription of proliferation-associated genes including GLI1, PTCH1, WNT1, forkhead box protein M1 (FOXM1), and CCND1 [86–88]. Moreover, SHH-induced signals are positively correlated with the expression of Snail and MMP9 and negatively with E-cadherin, suggesting that SHH signaling may be an important contributor to invasion and metastasis of HNSCC [89], by driving EMT. In tumor cells, SHH signaling cascades are aberrantly activated by genetic alterations, such as loss-of-function alterations in PTCH1, gain-of-function mutations in SMO (T241M, L412F, S533N, W535L, and R562Q), and amplification of the GLI1 or GLI2 genes [90, 91]. It has been shown that SHH signaling is activated in various CSCs, including breast cancer, liver cancer, gastric cancer, and HNSCC [92–95]. Interestingly, elevated levels of GLI1 are correlated with recurrence, lymph node metastasis and the worst prognosis in HNSCC patients [89, 95]. Detection of SHH pathway components, especially GLI1 and SHH, in HNSCC might represent promising targets for future anticancer therapeutic development in HNSCC. Another important player that is linked to CSCs in HNSCC is NOTCH1. NOTCH proteins are a family of heterodimeric transmembrane receptors composed of an extracellular domain, a transmembrane domain, and an intracellular domain. The intracellular domain translocates into the nucleus, where it modulates transcription *via* CBF1, Suppressor of Hairless, Lag-1 (CSL) transcription factor family [96, 97]. This complex activates the transcription of target genes including HES, HERP, and HEY which are involved in cellular differentiation [97]. Other NOTCH targets include the key cell cycle regulators cyclin D1, cyclin A, p21, and p27 [97]. Abnormal expression of NOTCH receptors has been observed in different types of malignant lesions. NOTCH signaling plays an essential role in a variety of stem cell processes in HNSCC, such as cell proliferation, differentiation, survival, and self-renewal [98]. In terms of anti-cancer therapeutics, NOTCH1 Inhibition by γ-secretase inhibitors reduces tumor growth and blocks CSC function in multiple tumors including breast cancer, brain tumor, and glioma [99]. Conversely, high levels of NOTCH1 correlate with increased resistance to cisplatin in HNSCC patients [100], while high expression of both NOTCH1 and JAG1 (a NOTCH 1 ligand) is associated with poor prognosis in HNSCC [101]. In addition, inhibition of NOTCH1 delays tumorigenesis, reduces CSC self-renewal and maintenance, and improves the efficacy of cisplatin and 5-fluorouracil by targeting CSCs in HNSCC [102]. In contrast, Grilli et al. [103] have revealed that NOTCH1 expression is positively correlated with non-recurrent disease, prolonged OS rates, and better prognosis in HNSCC patients. However, these authors did not determine or further investigate the mechanisms by which NOTCH signaling functions in an anti-tumorigenic manner.

**Figure 2:**
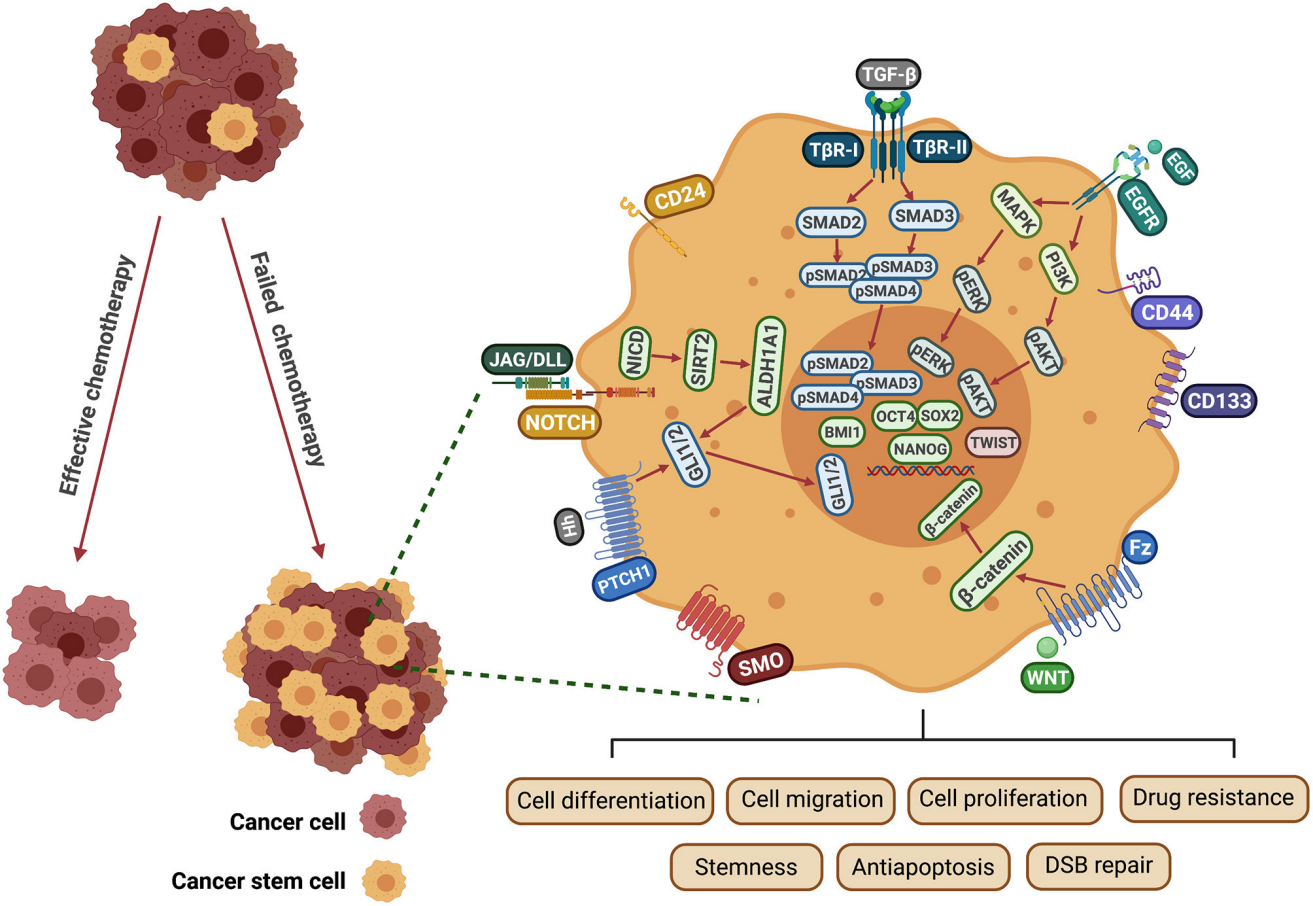
Mechanisms involved in CSC-induced drug resistance in HNSCC. EGF binds to the EGFR receptor tyrosine kinase resulting in activation and autophosphorylation of the receptor. This activates RAS and PI3K, triggering major signaling cascades that include MEK/ERK and PI3K/AKT. NOTCH-activated NICD1, upregulates the SIRT2/ALDH1A pathway. Hedgehog ligands (Hh) bind to Patched receptors and derepress the G-protein-coupled receptor (GPCR) SMO. Hh targets ALDH^+^ CSCs through GLI1 expression, which is regulated by ALDH1A1. WNT proteins bind to the Fz transmembrane receptor and the LRP 5/6 to form a functional complex. β-catenin then becomes uncoupled from the degradation complex and translocates to the nucleus to promote transcription of downstream targets. TGF-β is recognized by TβR1/2 resulting in the phosphorylation of SMAD 2/3 and formation of a SMAD 2/3/4 complex. These pathways are involved in regulating multiple biological functions of CSCs, including cell proliferation, migration, invasion, self-renewal and survival. EGF, epidermal growth factor; EGFR, epidermal growth factor receptor; Fz, frizzled; JAG, Jagged ligand; DLL, Delta-like ligand; DSB, double-strand break; NICD, intracellular domain of NOTCH protein; SMO, smoothened; MAPK, mitogen-activated protein kinase; PI3K, phosphoinositide 3-kinases; PATCH1, Protein patched homolog 1; TGF-β, transforming growth factor; TβR, transforming growth factor β receptor; ALDH1, Aldehyde dehydrogenase 1 family; BMI1, B lymphoma Mo-MLV insertion region 1 homolog; OCT4, Octamer-binding transcription factor 4; SOX2, Sex determining region Y-box 2; NANOG, Nanog homeobox.

The epidermal growth factor receptor pathway is one of the signaling cascades that control CSC differentiation and maintenance in HNSCC. EGFR is a transmembrane receptor tyrosine kinase that is activated by various ligands, including epidermal growth factor (EGF) and transforming growth factor-α (TGF-α), triggering activation of downstream signaling cascades such as PI3K/AKT, MEK-ERK, and phospholipase C signaling to control cell growth, survival, differentiation, angiogenesis, and invasion [104]. Overexpression of EGFR is associated with resistance to treatment and poor clinical outcomes with HNSCC patients [105]. Accumulating evidence indicates that EGFR plays a vital role in development of HNSCC stemness. It has been shown that CD44 interacts with EGFR to promote cell proliferation, migration and induces cisplatin resistance and apoptosis inhibition in HNSCC cells [106], however, these findings should be confirmed in tissues derived from HNSCC patients. Moreover, EGFR drives HNSCC metastasis by inducing glycolysis/EMT/CSC properties through a PI3K-dependent mechanism [107]. A recent study reported that EGFR activation induces SOX2 phosphorylation at Y277, inhibiting ubiquitination, and subsequent autophagic degradation of SOX2 in a human tongue SCC cell line [108]. In addition, EGFR overexpression is positively correlated with a higher functional proportion of ALDH^high^ CSCs in a human papillomavirus-16 (HPV-16)-positive cell line [109], however, the mechanism responsible for this is still unknown. WNT signaling is also critical for stem cell self-renewal and differentiation. Based on the involvement of β-catenin, WNT signaling can be divided into two pathways, namely canonical and non-canonical. WNT/β-catenin signaling pathway is activated by the binding of WNT protein to Frizzled (Fz) seven transmembrane receptor and the coreceptor lipoprotein receptor-related protein 5 or 6 (LRP 5/6) to form a functional complex [110]. Consequently, the β-catenin is uncoupled from the degradation complex and translocated into the nucleus to promote transcription of downstream targets such as cyclin D1 (*CCND1*), cyclooxygenase 2 (*COX2*), bone morphogenetic protein 4 (*BMP4*), matrix metalloproteinases 7 (*MMP7*), and *C-MYC* [111] (Figure 2). It has been established that WNT/β-catenin signaling plays a crucial role in maintaining the CSC phenotype in various types of cancer. A possible mechanistic explanation is that β-catenin interacts with CD44, and β-catenin inhibition by WNT decreases the expression of CD44 and OCT4 in HNSCC cells [112]. It is also reported that WNT pathway activation enhances the CSC proliferation rate and promotes stemness and sphere formation in HNSCC cell cultures, through upregulating the expression of SOX2 [113]. In addition, β-catenin plays a fundamental role in mediating cisplatin resistance by regulating DNA damage repair in HNSCC [114] (Table 1). These findings may form a basis for future studies aimed at developing novel strategies to combat drug resistance and disease recurrence in HNSCC.

**Table 1:** CSC markers in HNCs. Different markers for the identification and characterization of CSCs in HNCs, including HNSCC, ACC, MEC, and oral melanoma are listed.

**Types of CSC markers**	**Name of** **marker**	**Associated properties and functions**
Cell surface markers	CD44	Tumor initiation [9]. Enhancement of proliferation and migration, and apoptosis inhibition in HNC [106, 115]. • Overexpression is associated with regional lymph node metastasis, perineural invasion, increased loco-regional recurrence, increased resistance to radiotherapy, and decreased overall survival [31, 32, 35, 116].
	CD133	• Tumor sphere formation, tumorigenicity, and chemoresistance in HNSCC [42]. • Acceleration of angiogenesis, clonogenic and tumorigenic abilities in melanoma [117–119]. • Positive correlation with poor overall survival in HNSCC patients [44, 120].
	CD10	Associated with tumor size, histological grade of malignancy, local recurrence, and therapeutic resistance in HNSCC [36, 37].
	ABCB5	• Promotes melanoma metastasis by activating the NF-κB cascade [121]. • Self-renewal and tumor initiation [122].
Metabolic marker	ALDH1	• Tumorigenic phenotype, self-renewal and stemness properties, and resistance to radiotherapy and chemotherapy in HNSCC [49]. • Sphere-forming, tumorigenic, and metastatic abilities in ACC [123]. • Colocalization with Snail and MMP-9 and induction of EMT-related genes in HNSCC [49, 52]. • Strong correlation with nodal metastasis and cisplatin resistance [124, 125].
Pluripotency markers	BMI1	• Self-renewal, colony formation, migration, and invasion in HNSCC [126]. • Associated with overexpression of the EMT-related transcription factors Snail and Slug in ACC [127]. • Strong correlation with advanced stages, aggressive clinicopathological behaviors, stem cell-like properties, drug resistance, and poor prognosis in HNSCC [82, 128].
	SOX2	• Known to complex with OCT4 and control downstream embryonic genes including NANOG [73]. • Involved in cell proliferation, migration, invasion, stemness, tumorigenesis and anti-apoptosis, and chemoresistance in HNSCC [68]. • Associated with surviving expression in ACC patients [129]. • Correlate positively with radiochemoresistance and poor prognosis in HNSCC patients [68, 71].
	OCT4	• Role in the regulation of epithelial–mesenchymal transition through increasing expression of N-cadherin and Slug [77]. • Observed in metastatic lymph nodes and recurrent tumors from OSCC patients [10]. • Correlated with poor survival and considered as an independent prognostic marker of HNSCC progression [58, 65].
	NANOG	• Overexpressed in HNSCC CSCs [130]. • Associated with tumor transformation, tumorigenicity, and metastasis in HNSCC [58]. • Correlated with histopathological features of MEC including perineural invasion and desmoplasia [131]. • Correlated with poor differentiation status, chemoresistance, and poor prognosis [77, 132].
Self-renewal pathways	SHH	• Promote tumor growth and angiogenesis in HNSCC [89]. • Correlated with the expression of Snail and MMP9 [89]. • Correlated with recurrence, lymph node metastasis, and the worst prognosis in HNSCC patients [89, 95].
	NOTCH	• Involved in cell proliferation, differentiation, survival, self-renewal, and tumorigenesis [98, 99]. • Correlated with increased resistance to cisplatin and poor prognosis in HNSCC patients [100].
	EGFR	• Involved in cell proliferation, migration, cisplatin resistance, and apoptosis inhibition in HNSCC cells [106, 133]. • Promotes the stemness and progression of oral cancer [107, 108]. • Stabilizes and induces Snail-dependent EMT in ACC [134]. • Associated with resistance to treatment and poor clinical outcomes with HNSCC patients [105].
	WNT	Involved in CSC proliferation, sphere formation, and cisplatin resistance in HNSCC [113, 114].

## Characterization Of Oral Non-SCC Cancer Stem Cells

### Salivary Gland Cancers

Salivary gland cancers are rare, accounting for only 2–6% of head and neck cancers. In spite of this, however, they constitute a significant public health issue [135]. The most two common salivary gland malignancies are mucoepidermoid carcinoma (MEC) and adenoid cystic carcinoma (ACC). MEC occurs in both the major and minor salivary glands and represents ~30% of malignant salivary gland tumors [136]. The existence of CSCs has been identified functionally in salivary gland MEC, and ALDH^+^ CD44^+^ MEC cells exhibit self-renewal and multipotency, and are highly tumorigenic [137]. In addition to these markers, BMI1, OCT4, and NANOG were highly expressed in MEC cells [131]. Importantly, the expression of OCT4 and NANOG was correlated with histopathological features of MEC including perineural invasion and desmoplasia [131]. Moreover, a combination of three cancer stem cell markers - CD44, CD133 and SOX2 - was suggested to be a powerful and practicable prognosticator for patients with MEC of minor salivary glands [138]. Additionally, there was a positive correlation between CD44 and vimentin (a marker of EMT) expression level, and the levels of both CD44 and vimentin are associated with MEC tumor grade [115]. A previous study has reported that suberoylanilide hydroxamic acid (SAHA), an inhibitor of histone deacetylases (HDACs), was capable of disrupting CSCs in MEC cell lines and sensitizing tumor cells to cisplatin treatment, emphasizing the role of MEC CSCs in the well-recognized resistance of salivary gland tumors to chemotherapy [139].

ACC is the second most common malignant salivary gland tumor, accounting for 10–25% of the total, and it is the most common histological subtype observed in patients with distant metastatic disease [140]. Previously, it has been reported that ALDH1 was expressed in stromal cells in the majority (63%) of ACCs, although, the pattern of ALDH1 expression did not affect survival of ACC patients [141]. Another study revealed that ALDH^+^ ACC cells generate the phenotypic diversity of the initial tumor, and have robust sphere-forming, tumorigenic, and metastatic abilities [123]. In addition, high expression of BMI1 was observed in ACC samples with distant metastases as compared to those with negative status, and there was a significant correlation with poor clinical outcome [142]. It has also been shown that BMI1 upregulation is associated with overexpression of the EMT-related transcription factors Snail and Slug (SNAI2) in ACC [127]. SOX2 is also highly expressed in ACC, being tightly associated with the clinical outcome of ACC and, therefore, it has been suggested to have utility as a prognostic marker in this tumor type [143]. Furthermore, there was a significant correlation between SOX2 and Survivin (IAP5; an anti-apoptotic protein) in ACC patients, and the levels of these were significantly higher than in the control group [129]. EGFR is another important player that is commonly upregulated in ACC and has a role in CSC self-renewal. As mentioned above, EGFR signaling holds a pivotal role in self-renewal and maintenance of stem cells. Some previous studies have reported overexpression of EGFR in a minority of ACC cases, which suggests that there is minimal involvement of this pathway in ACC pathogenesis [144, 145], although, overexpression is not necessarily indicative of pathway activity. However, other investigators have reported that 85% of ACC tumors express high levels of EGFR and that 10% of cases express mutated (active) EGFR [146, 147]. The wide variation in results could be explained, at least in part, by the difference in specificity and sensitivity of the techniques used to detect EGFR. One of the proposed mechanisms of EGFR maintenance of stemness is that it leads to stabilization of Snail, with EMT induction [134]. Therefore, analysis of downstream mediators such as Snail or other EMT regulators, or of pathway activation, may provide a more definitive answer. Another study has recently reported that blocking EGFR with erlotinib increases the activation of NOTCH1 signaling, leading to induction of stem cell-like properties [148]. Furthermore, multiple other ligands, including transforming growth factor-α (TGF-α), can activate EGFR; thus, overexpression of such ligands could be implicated in ACC pathogenesis [145]. Taken together, these results suggest that EGFR may utilize multiple mechanisms to promote CSC self-renewal and stemness in ACC (Table 1).

### Oral Melanoma

Oral melanoma is a rare (only 0.5% of oral malignancies) and aggressive malignancy with a very poor prognosis [149]. It presents most frequently in the hard palate and alveolar gingiva. The prognosis for patients with these tumors is poor, with a 5-year survival rate estimated at between 20 and 38% [150]. As a result of the high degree of plasticity of this cancer, and the existence of multiple mechanisms that lead to melanoma progression, the existence of a unique and specific biomarker signature for melanoma stem cells is still controversial. Among the surface markers commonly used to identify melanoma stem cells are CD133, CD271, ABCB5, and ALDH1A [151]. CD133 has been associated with CSCs in different tumors including melanoma [151, 152]. A previous study indicated that CD133^+^ CSCs isolated from the metastatic melanoma cell line D10 significantly induced and accelerated initial angiogenesis compared to CD133^−^ cells [117]. Expression of CD133 has also been reported to be high in metastatic melanoma compared to primary melanoma, and CD133^+^ melanoma cells have higher clonogenic and tumorigenic abilities compared to CD133^−^ cells [118, 119]. In contrast, a meta-analysis evaluated 299 melanoma cases from five studies for expression levels of CD133 and reported low power to detect a significant association between CD133 expression and melanoma progression [153], suggesting that CD133 might not be an appropriate biomarker in identifying melanoma CSCs. However, in spite of this low power, the authors found 47.9% of cases had high CD133 expression, indicating a positive correlation between CD133 expression and disease progression. High variability was observed in the expression of CD271 as a CSC marker and some controversy still exists with regard to its function in melanoma stem cells. Nevertheless, other studies have suggested that CD271 may contribute to the aggressive nature of melanoma cells and associated chemoresistance [154, 155]. Another functional driver of melanoma aggressiveness and drug resistance is ATP-binding cassette sub-family member 5 (ABCB5), which promotes drug efflux in cancer cells [156]. One study found no correlation between ABCB5 expression and tumor-initiating capacity [157]. However, in a more recent report, ABCB5 was found to enhance tumorigenic ability and promote melanoma metastasis by activating the NF-κB cascade [121]. In addition to these markers, melanoma CSCs show high ALDH activity, especially ALDH1A1 and ALDH1A3 isozymes. ALDH^+^ melanoma cells were found to be more tumorigenic and more resistant to chemotherapeutic agents than ALDH^−^ cells [158]. Moreover, xenografts from ALDH^+^ melanoma cells displayed superior self-renewal compared to xenografts from ALDH^−^ cells [159]. However, a recent study reported that both ALDH1A1 and ALDH1A3 correlated with favorable prognosis in metastatic BRAF wild-type and BRAF-mutant melanoma, respectively [160]. Other intracellular proteins of likely importance in melanoma stem cells are SOX2 and KLF4. As mentioned above, they are pluripotency markers and their high expression in melanoma cells promotes reprogramming toward a stem cell phenotype, inducing cell proliferation and drug resistance, and inhibiting apoptosis, [161, 162]. Recently, it has been found that the expression of OCT4, SOX2, and KLF4 was high in CSC subpopulations within the tumor nests and peritumoral stroma of head and neck metastatic malignant melanoma [163]. Another important player is c-MYC, another transcription factor regulating pluripotency and self-renewal of ESCs. For many years, c-MYC has been implicated in the development of numerous cancer types, including melanoma. It has been demonstrated that c-MYC overexpression is associated with tumor progression and is preferentially expressed in metastatic melanoma [164, 165]. Additionally, high c-MYC expression in mucocutaneus melanoma correlates with immune evasion and tumor invasiveness, hence, its association with poor prognosis and survival [164, 166]. In addition, mutations in KIT (the receptor for stem cell factor, SCF) have been found in ~15% of mucosal melanomas [167]. A previous study has indicated that KIT signaling is critical for the proliferation and migration of melanoma cells, raising the possibility that this might be a promising therapeutic target in patients with aggressive oral melanoma [168] (Table 1).

## Targeting and Challenges in HNSCC Cancer Stem Cell-directed Therapy

**Figure 3:**
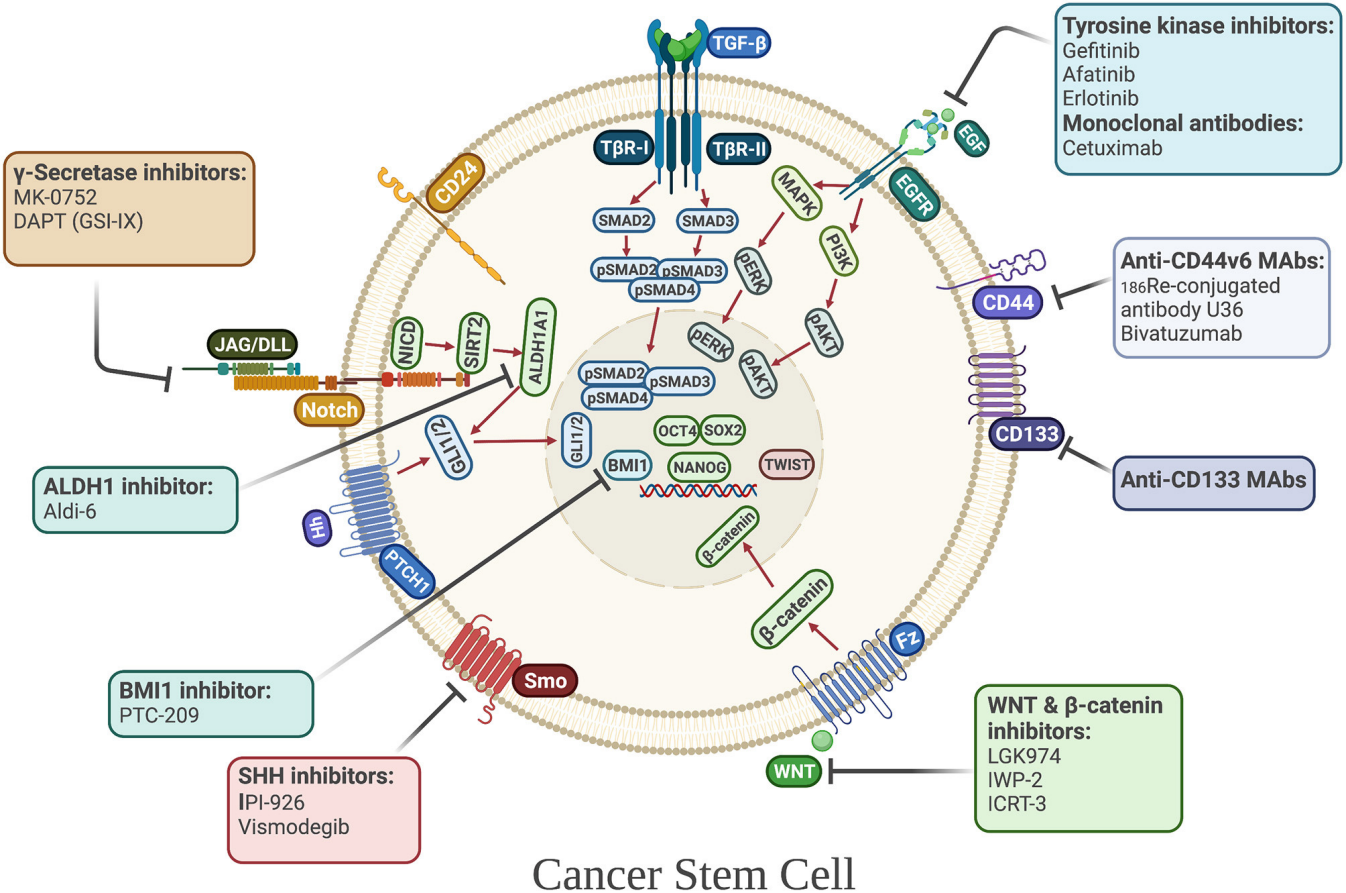
Cancer stem cell-directed therapies in HNSCC. Selected anti-CSC drugs currently under clinical investigation. Their mechanisms of actions include targeting CSC-associated surface markers and CSC-associated signaling pathways, including developmental pathways, that regulate the maintenance, and survival of CSCs.

Despite the advances in targeted therapy for HNSCC, early studies from clinical trials have reported limited efficacy with monotherapy compared to conventional therapies. Thus, new therapeutic strategies targeting CSCs are under development to be used in combination with conventional non-targeted therapies to prevent tumor relapse, metastasis, and to combat chemoresistance (Figure 3).

### Targeting Self-Renewal Pathways

To date, one of the most promising strategies for targeting HNSCC CSCs is blocking the key self-renewal signaling cascades, such as those regulated by EGFR, NOTCH, WNT, and SHH. Inhibition of EGFR is being employed in advanced and recurrent HNSCC treatment. It has been determined that gefitinib (a tyrosine kinase inhibitor) preferentially targets CSCs, eliminating tumor regrowth, and increasing sensitivity to cisplatin in nasopharyngeal carcinoma [169]. Moreover, blocking EGFR with gefitinib reduces the expression of c-MYC and NANOG, essential factors for reprogramming of induced pluripotent stem cells [169]. Interestingly, treatment with the anti-EGFR antibody, cetuximab, induces CSC differentiation, and inhibits radiochemoresistance of CSCs in HNSCC [170]. Another study has indicated that afatinib (a second-generation tyrosine kinase inhibitor) reduces the self-renewal and invasive properties of HNSCC CSCs in culture by downregulating CD44 and OCT4, inhibiting tumor sphere formation and growth, and inducing radiosensitization [171]. In contrast, in a phase III study, monotherapy with gefitinib did not improve the OS in patients with recurrent and/or metastatic HNSCC [172]. However, the combination of EGFR inhibitors with cisplatin or radiotherapy shows improved response compared to monotherapy with erlotinib or cetuximab, raising the possibility that resistance of tumors to EGFR inhibitor monotherapy could be related to the functional heterogeneity of CSCs in advanced HNSCC [170, 173]. As NOTCH, WNT, and SHH signaling play essential roles in CSC maintenance in HNSCC [174], these pathways are considered as attractive targets for treatment of recurrent/metastatic HNSCC. A preclinical study found that blocking NOTCH1 with the γ-secretase inhibitor, DAPT, modifies the HNSCC CSC phenotype, reducing chemoresistance in tissues post chemotherapy, and also lymph node metastasis [102]. In a non-randomized phase I trial, the combination of a NOTCH inhibitor, MK-0752, with an mTOR inhibitor, ridaforolimus, showed clinical activity in metastatic HNSCC; however, a high number of adverse effects were reported [175]. For WNT signaling, the inhibitor of β-catenin responsive transcription (ICRT-3) arrests the cell cycle and decreases the motility of HNSCC cells [176]. Other preclinical studies have focused on inhibition of Porcupine, a membrane-bound acyltransferase, and found that Porcupine inhibitors such as LGK974 and IWP-2, induce apoptosis, inhibit cell migration and reduce the expression of CSC markers in HNSCC [177, 178]. A similar pattern is also seen with SHH inhibitors, as one study has shown that treating EGFR inhibitor-resistant HNSCC cells with the SHH inhibitor IPI-926 reduces tumor growth and blocks tumor recurrence in patient-derived HNSCC xenografts [179]. Moreover, vismodegib, a SHH pathway inhibitor, decreases expression of GLI1 and Survivin, and promotes radiation-induced DNA damage in HNSCC cells [180]. Another study investigated the dual targeting of EGFR and SHH pathways and found a reduction of cell proliferation and colony forming ability of HNSCC cells [181]. Furthermore, a pilot study testing cetuximab and IPI-926 in patients with recurrent and/or metastatic HNSCC has revealed good tolerability and anti-tumor efficacy [182], indicating that combinational therapy blocking EGFR and SHH might improve the clinical outcomes for HNSCC patients.

### Targeting Metabolic and Cell Surface Markers

The markers used to identify and enrich CSCs may have potential as targets for HNSCC therapy. Among the first reports involving therapeutic targeting of CD44, one study investigated the effect of ^186^Re-conjugated U36 antibody against the splice variant CD44v6, which was well-tolerated and showed initial promise. However, long-term disease stabilization was only observed in one of 13 patients [183]. Another clinical study indicated that the anti-CD44v6 monoclonal antibody BIWA 4 (bivatuzumab) has antitumor effects and disease stabilization was observed in patients with recurrent locoregional and/or metastatic HNSCC [184]. Labeling BIWA 4 with both a near-infrared fluorescent dye (IRDye800CW) and a radioactive label (Indium-111) was used recently to detect xenografted HNSCC cells, raising the possibility that targeting CD44v6 might be useful for fluorescence-guided surgery [185]. Anti-CD133 agents have also been investigated as targeted anti-CSC therapy in HNSCC. A preclinical study revealed that CD133 knockdown decreases the percentage of side-population CSCs, diminishes tumor growth *in vivo*, and overcomes chemoresistance [43]. Another study in which a bacterial toxin (cytolethal distending toxin) was conjugated to an anti-human CD133 monoclonal antibody demonstrated inhibition of CD133^+^-induced cell proliferation in cultures of established HNSCC cell lines [186]. Another approach has favored targeting the drug-detoxification enzyme ALDH1A1, and a strong correlation was found between high levels of ALDH1A1 expression and cisplatin resistance that were reversible by an ALDH1A1 inhibitor which blocks CSC-related activity [124]. In addition, treatment with a novel small molecule inhibitor of ALDH3A1 (Aldi-6) decreased cell viability, and the combination of Aldi-6 and cisplatin caused a profound reduction of cell viability and a greater reduction in tumor size *in vivo* [187]. Taken together, it seems that combinational therapy using CSC inhibitors together with standard chemotherapy agents could hold significant promise as a future therapeutic strategy for HNSCC.

### Targeting Stem Cell Factors

Another potential therapeutic target to eradicate CSCs is the transcription factor NANOG. Targeting NANOG in combination with cisplatin suppressed stem cell properties of HNSCC cells and enhanced apoptosis and chemosensitivity [188]. Another recent preclinical study found that the combination of NANOG inhibition and radiotherapy produced an additive effect with a decrease in cell viability, stemness properties, and radiotherapy resistance of CD44^+^ HNSCC cells [76]. In the same study, the authors investigated the involvement of ERK1/2-NANOG signaling on tumor growth and metastasis and found that ERK1/2-NANOG inhibition may reverse CSC phenotypes and have potential to reduce tumor progression and metastasis in HNSCC patients. Additionally, combined treatment with 4SC-202, a novel selective class I histone deacetylase (HDAC) inhibitor, and INK128, a selective mTORC1/C2 inhibitor, synergistically inhibits SOX2 expression and cell growth, and reduces ALDH1^+^ CSCs and sphere-forming ability of HNSCC cells [189], suggesting that combined HDAC and mTORC1/C2 inhibition selectively targets the self-renewing capacity of CSCs and is more effective and promising than conventional chemotherapy. Furthermore, knockdown of BMI1 was shown to increase the sensitivity of HNSCC cells to radio/chemotherapy in HNSCC-ALDH1^+^ cells [190]. Other preclinical studies have revealed that the BMI1 inhibitor, PTC-209, inhibits cell proliferation and migration, eliminates lymph node metastases, and reduces colony formation as well as the percentage of ALDH^+^ cells [82, 191]. Recently, it has been demonstrated that combination therapy with PTC-209 augmented PD1 immune checkpoint blockade and eliminated BMI1^+^ CSCs by inducing tumor cell-intrinsic immunity, resulting in the inhibition of metastasis and relapse of HNSCC *in vivo* [192]. Although, monotherapy with the BMI1 inhibitor suppressed HNSCC growth and metastasis, it was not as effective as combination therapy with anti-PD1.

## Targeting and Challenges in Oral Non-SCC Cancer Stem Cell-Directed Therapy

### Targeting Salivary Gland CSCs

Malignant salivary gland tumors are characterized by frequent local recurrence and distant metastasis, and no satisfactory method has been determined to treat these lesions. Surgery and radiotherapy are reserved to treat localized disease, while chemotherapy is necessary to manage recurrent and/or metastatic tumors, although, no chemotherapy regimen has yet been proven to improve OS [193–197]. Due to these unfavorable outcomes, various targeted agents have been investigated as potential new treatments for salivary gland carcinomas. As mentioned above, EGFR overexpression occurs in a high proportion of ACC cases, and varies markedly among different histotypes, making it a potentially attractive therapeutic target. A phase II study revealed no correlation between EGFR expression/status and response to cetuximab, although, the majority of ACC patients obtained disease stabilization [198]. Consistent with this study, another clinical trial observed a response with lapatinib treatment with prolonged tumor stabilization of more than 6 months in recurrent or metastatic salivary gland carcinoma patients, suggesting that targeted therapy with anti-EGFR therapeutics may improve the clinical outcomes of patients suffering from malignant salivary gland tumors [199]. However, another phase II study demonstrated no objective responses with gefitinib as a monotherapy in patients with advanced salivary gland cancer [200]. Continued work toward the development of predictive biomarkers to inform better treatment options for patients is needed to improve clinical outcome in ACC. A case report indicated that the combination of radiotherapy and cetuximab was well-tolerated and showed a complete remission in a patient with recurrent high grade MEC [201], suggesting that, as for SCC, the combination of anti-EGFR and conventional therapies could be more effective than anti-EGFR monotherapy to manage salivary gland carcinomas. However, further clinical investigation is needed to determine the impact of combinational therapy of anti-EGFR and conventional therapies on patient with MEC. NOTCH1 has also been tested as a therapeutic target for salivary gland CSCs. Genetic reduction of NOTCH1 using RNA interference suppresses proliferation, migration, and clonogenic growth of ACC cells in culture, and reduces the number of metastatic nodules in the lungs of immunodeficient mice bearing ACC xenografts [202]. In an open-label phase I trial, LY3039478, a selective oral NOTCH inhibitor, was well-tolerated; however, no partial or complete responses were observed, although, the majority of the patients (58%) with ACC achieved stable disease [203]. Additionally, Brontictuzumab, a humanized monoclonal antibody that binds to the NOTCH1 juxtamembrane negative regulatory region, was found to have antitumor efficacy in a subcohort of patients with NOTCH-activated ACC [204]. Notably, cisplatin treatment increased the proportion of ALDH^high^/CD44^high^ cells, the number and size of spheroids and BMI1 expression in MEC cell lines. However, the combination of cisplatin and the mTOR inhibitor, temsirolimus, decreased the expression of BMI1, inhibited mTOR signaling, reduced spheroid formation, and decreased the CSC fraction in MEC patient-derived xenograft tumors [205]. Moreover, the combination of cisplatin and the histone acetyltransferase inhibitor, vorinostat, reduced ALDH expression, and CSC numbers, as well as increasing chemosensitivity of MEC cells to cisplatin [139]. Overall, it is likely that combination treatment of targeted therapy with conventional agents might hold promise as a path to improve clinical outcomes. Currently, several clinical trials of targeted therapy for salivary gland malignancies are ongoing.

### Targeting CSCs of Mucosal Melanoma

Previous studies have demonstrated that melanoma cells that have attained a chemoresistant phenotype express multiple stem cell markers, as mentioned above, suggesting that melanoma CSCs may drive the phenotype of these tumor and, thus, represent a very attractive target for novel treatment. One potential therapeutic target is the receptor tyrosine kinase KIT. In phase II clinical trials, the KIT inhibitor imatinib has been evaluated in patients with mucosal melanoma harboring KIT mutations, with an overall disease control rate of ~50%. Importantly, around half of patients with KIT mutations responded to imatinib treatment while none harboring KIT amplification showed a similar response [206]. Another study tested the impact of blocking the ABCB5 transporter on recurrent melanoma, but found that knockdown of ABCB5 did not re-sensitize BRAF inhibitor-resistant melanoma cell lines, in spite of the fact that ABCB5 is highly expressed in malignant melanoma-initiating cells [207]. This suggests that ABCB5 may not be a useful therapeutic target for patients with BRAF inhibitor-resistant melanoma. Another important player that mediates stemness properties in mucosal melanoma, and therefore, might be a promising target, is ALDH. In a recent preclinical study, activated nifuroxazide, which belongs to the family of 5-nitrofuranes, specifically targets ALDH1^high^ melanoma cells by reducing the survival of melanoma CSCs [208]. In the same study, it was shown that combination of a BRAF inhibitor and a MEK inhibitor led to an adaptive increase in ALDH^high^ subpopulations in a subset of melanoma cell lines. However, in phase III trials, combination of the BRAF inhibitor, dabrafenib, and the MEK inhibitor, trametinib, resulted in improved progression-free survival, and overall response and survival rates compared to dabrafenib monotherapy in untreated patients who had metastatic melanoma with BRAF V600E or V600K mutations [209]. Furthermore, the combination of the BRAF inhibitor, vemurafenib, and the MEK inhibitor, cobimetinib, also provided a significant benefit compared to vemurafenib alone [210], suggesting that dual targeting may provide better clinical outcomes for metastatic melanoma. Moreover, it also indicates that caution is advised when translating preclinical study results, carried out with cell lines, to patient populations. Additionally, dual targeting with the Bcl-2 inhibitor, ABT-737, and the γ-secretase Inhibitor, GSI, revealed a high efficiency in reducing the cell viability, disrupting colony formation, decreasing ALDH^+^ cells, and inhibiting the self-renewal of melanoma CSCs [211], indicating that this combinational therapy might be another promising strategy to address treatment relapse of malignant melanoma.

## Conclusions

Even though, substantial progress has been made recently in the development of different therapeutic strategies to treat HNC, including targeted therapy, unfavorable clinical response is still a common problem due to therapeutic resistance. Conventional therapies have limited therapeutic effects against the CSC subpopulation. CSC-targeted therapy has been found to be a promising strategy to sensitize resistant tumor cells, eliminate residual tumor-initiating cells, and prevent disease relapse, resulting in improved OS. Multiple CSC biomarkers have been identified that are correlated with poor prognosis, and are attractive targets for therapy. CSC-targeted monotherapies are frequently associated with development of resistant phenotypes or lack of clinical response. Combinatorial therapies address different facets of the CSC phenotype, and hold promise to improve HNC patient outcomes. However, there is a pressing need to discover more specific CSC properties and their druggable targets, as well as distinguishing patient-specific CSC subsets, in order to increase specificity of treatments and reduce serious side-effects experienced by cancer patients. As another point of note, the utility of CSC-targeted therapy could be complicated as a result of serious adverse effects due to targeting pathways that are critical for normal tissue stem cells. Thus, further clinical research to determine the therapeutic value of targeting CSC markers, as well as a more comprehensive understanding of the nuances of cancer stem cells, would help to improve the clinical outcomes of HNC patients.

## Author Contributions

LS conceived the idea, wrote and revised the manuscript, and is solely responsible for its content.

## Conflict of Interest

The author declares that the research was conducted in the absence of any commercial or financial relationships that could be construed as a potential conflict of interest.
